# A Review of Repurposed Cancer Drugs in Clinical Trials for Potential Treatment of COVID-19

**DOI:** 10.3390/pharmaceutics13060815

**Published:** 2021-05-30

**Authors:** Bárbara Costa, Nuno Vale

**Affiliations:** 1 OncoPharma Research Group, Center for Health Technology and Services Research (CINTESIS), Rua Dr. Plácido da Costa, 4200-450 Porto, Portugal; b.c.211297@gmail.com; 2 Department of Community Medicine, Health Information and Decision (MEDCIDS), Faculty of Medicine, University of Porto, Al. Prof. Hernâni Monteiro, 4200-319 Porto, Portugal

**Keywords:** drug repurposing, COVID-19, cancer, pandemic, vaccination

## Abstract

The pandemic of the coronavirus disease 2019 (COVID-19) represents an unprecedented challenge to identify effective drugs for prevention and treatment. While the world’s attention is focused on news of COVID-19 vaccine updates, clinical management still requires improvement. Due to the similarity of cancer-induced inflammation, immune dysfunction, and coagulopathy to COVID-19, anticancer drugs, such as Interferon, Pembrolizumab or Bicalutamide, are already being tested in clinical trials for repurposing, alone or in combination. Given the rapid pace of scientific discovery and clinical data generated by the large number of people rapidly infected, clinicians need effective medical treatments for this infection.

## 1. Introduction

The coronavirus disease 2019 (COVID-19) pandemic, caused by the severe acute respiratory syndrome coronavirus-2 (SARS-CoV-2), has caused catastrophic damage to human life. Since December 2019, the pandemic has spread worldwide and still is ongoing. SARS-CoV-2 primarily infects the upper and lower respiratory tract; however, it can also affect other vital organs. Most people recover from the acute phase of the disease, but some people continue to experience a range of effects for months after recovery. Clinical management is currently focused on supportive care and prevention and control of complications such as acute respiratory distress syndrome (ARDS) [1].

Although the world’s attention is understandably centred on reports of COVID-19 vaccine updates, from supply to administration, the need for treatments cannot be overlooked, as vaccination cannot protect everybody and as infection overwhelms hospitals and nursing homes. When we compare COVID-19 to the common flu, which is routinely targeted and has readily available and effective vaccines, we can see that no vaccine is ideal. Therefore, flu medications are still in high demand to avoid hospitalization and save lives. While the rise of new variants of COVID-19 threatens the efficacy of the available vaccines, it is critical that we must continue researching therapies to minimize hospitalization and cure COVID-19. The world health organization created (WHO) guidelines on using vaccines and antivirals during influenza pandemics to address the shortage of vaccines and antivirals [2]. Demonstrating that with therapy, people can live longer and gain control over the pandemic’s curse, as the likelihood of people becoming ill and spreading the disease decreases. Therapeutics also can be used as prophylactics to prevent hospitalizations and severe cases of the disease.

The food and drug administration (FDA) granted emergency use authorization to two monoclonal antibody treatments for non-hospitalized adults and children over the age of 12 who have mild to moderate COVID-19 symptoms, who are at risk for developing severe COVID-19 or being hospitalized for it. Regeneron’s casirivimab and imdevimab combo and Eli Lilly’s bamlanivimab and etesevimab combination are the two treatmentsPrior approval for the single use of bamlanivmab to treat COVID-19 was withdrawn in April 2021 due to new data revealing minimal efficacy [3]. While these medications can be beneficial, the need for intravenous administration (IV) requires a visit to a clinic or hospital immediately after symptoms appear, which limits their use.

Consequently, effective therapies, which are available to anyone who needs them, must work with various populations and ensure that the responses to the pandemic are globally successful and inclusive. Having both important tools in our arsenal would ensure that most of the population is shielded from the severe effects of COVID-19. However, the development of novel antiviral drugs needs long-term investigation in clinical trials. Therefore, the benefit of repurposing drugs to justify off-label usage is linked to the established safety profile. However, it may vary depending on the disease and the consolidated data on pharmacodynamics, pharmacokinetics and efficacy in phase I–IV trials [4,5]. Some host cell targets that interfere with the viral growth cycle, such as kinases, are commonly shared in the mechanisms of multiple viral infections and other conditions such as cancer, indicating the possibility of translating information through medical disciplines and disease models [6].

Several anticancer compounds were investigated as possible future drugs for COVID-19, among the thousands of coronavirus drugs studied. This article includes anticancer drugs that have already been approved or are being fast-tracked by regulatory authorities, supported by published evidence and used to treat the treatment of cancer patients. In times of crisis, such as COVID-19, drug repurposing is a valuable technique because it provides quick access to agents with not only accessible safety data but also defined manufacturing lines and supply chains, which facilitates the process of discovery. The major limitation of the use of repurposed therapeutics is associated with dosage regimens. Most of the time, effective concentrations needed for antiviral activity are often higher than those clinically attainable under the approved regimens [7].

Drug repurposing is not a reason for designing low-quality clinical trials or emphasizing the bias of early outcomes and uncontrolled cohorts, and it is desirable to use molecules with a specified safety profile. In addition, these therapies have early-and late-phase data on toxicity and complications management, which are especially helpful in the setting of a pandemic versus novel therapies. Several antineoplastic agents have the potential to improve COVID-19 outcomes by using the exact mechanisms and targets used in cancer treatment [8]. These targets are primarily associated with inhibiting cell division, regulating inflammation, and modulating the host-tumor microenvironment.

## 2. Materials and Methods

Using the OpenData Portal [9] was possible to research COVID-19-related drug repurposing data and experiments for all approved drugs. From this portal were only selected the anticancer drugs tested. Then was searched on the database of clinicaltrials.gov [10], the list of anticancer drugs tested on COVID-19 to see which were listed in clinical trials (20 May 2021). Additionally, searching through the site of European Pharmaceutical Review [11], in the news section, we were able to find more information about repurposed drugs and clinical trials for COVID-19. In the end, it was possible to formulate an updated list of anticancer drug candidates for COVID-19 treatment.

## 3. Viral, Host and Immune Targets in COVID-19

Antiviral therapy and prevention approaches are focused on (a) inhibiting the replication of the viral genome by either preventing the virus from entering the host cells or suppressing one or more phases of replication; (b) boosting the immune system and producing a type of antiviral memory via vaccination; (c) injection of antiviral antibodies generated in the plasma [12].

SARS-CoV-2 replicates similarly to other Coronaviridae viruses. Coronaviruses can infect the host through both endosomal and non-endosomal (cell surface) routes. The viral protein kinases and their associated signaling cascades have now been targeted in order to reduce coronavirus replication, particularly SARS-CoV-2. The virus can enter the cells via endocytosis or plasma membrane fusion through the interaction between the Spike (S) protein of the virus and angiotensin-converting enzyme 2 (ACE2) and transmembrane protease serine 2 (TMPRSS2) at the target cell [12,13].

After receptor-mediated endocytosis of the virus into the host cells, the virus releases the viral genome (single-stranded positive RNA) and uses the host ribosome to translate into viral polyproteins. Viral proteinases 3CLpro and PLpro cleave viral polyproteins into effector proteins (see Appendix A). RNA-dependent RNA polymerase, in turn, synthesizes a full-length negative-strand RNA template, which is used to make more viral genomic RNA. The viral genome then is synthesized by genomic replication, and four essential structural viral proteins (nucleocapsid (N), spike (S), membrane (M) and envelope (E)) are produced by transcription and translation [14]. The N protein binds genomic RNA, while S, M and E proteins are integrated into the membrane of the endoplasmic reticulum (ER), forming ERGIC—endoplasmic reticulum-Golgi intermediate compartment (also referred to as a vesicular-tubular cluster). The assembled nucleocapsid with helical twisted RNA is encapsulated into the ER lumen, viral progeny is transported by the ERGIC toward the plasma membrane of the host cell, and finally, the daughter virus is released by exocytosis [15].

The SARS-CoV-2 infection activates both innate and adaptive immune responses in the host. Patients with severe COVID-19 have a lower number of natural killer (NK) cells and a higher level of the C-reactive protein. The early failure of antiviral immunity during SARS-CoV-2 infection is correlated with a significant decrease in total T cells and NK cells [16].

Exploring potential clinical targets for COVID-19 attenuation is critical for long-term COVID-19 treatment.

## 4. Similarities of Cancer Immune Response and COVID-19

Cancer treatment is still a major challenge, but tremendous progress in anticancer drug discovery and development has occurred in the last few decades. The spent decades developing drugs for cancer-induced inflammation, immune dysfunction, and vascularization provided us with a number of drug options that could be useful in the treatment of other diseases.

Patients affected by COVID-19 also display inflammation, immune dysfunction and vascular syndrome dysfunction [17].

Evidence suggests that the immune response to SARS-CoV-2 can play different roles: dysregulated immune responses in critically ill patients with COVID-19 is reflected by lymphopenia, mainly affecting CD4+ T cells, including effector, memory, and regulatory T cells, and decreased IFN-γ expression in CD4+ T cells. Exhaustion of cytotoxic T lymphocytes, activation of macrophages, and a low human leukocyte antigen-DR expression on CD14 monocytes has been noted in patients with COVID-19 [18]. These similarities led scientists to consider anticancer therapy for the management of COVID-19 [8].

Furthermore, the homeostasis maintained by the vascular endothelium in health is affected by COVID-19 infection. In clinical studies, patients with COVID-19 have higher levels of fibrinogen, fibrin degradation products, and D-dimer, which appear to be related to disease severity and thrombotic risk [17]. Since the susceptibility to thrombotic events tends to be, at least in part, linked to inflammation and activation of the innate immune system that can cause systemic coagulation pathways. Therefore, the counterparts between the mechanisms of immunotherapy-related toxicities and the COVID-19 cytokine storm must be well considered in order not to affect the efficiency of the reused drug and increase the risk of the disease.

## 5. Repurposing Anticancer Drugs against COVID-19

The drug repurposing approach puts the drug discovery process on a fast track. COVID-19 researchers’ attention to its potential growth is wider in a range of different scientific fields. Due to the availability of in-vitro and in-vivo screening data, chemical optimization, toxicity studies, bulk manufacturing, formulation development and pharmacokinetic profiles of FDA-approved drugs, drug development cycles are shortened as all these critical steps can be bypassed [7,8]. In addition, there is no need for larger investments and repurposed drugs are proven to be safe in preclinical models, thus lowering the attrition rates as well. The main advantage of drug repurposing is associated with the established safety of the known candidate compounds. The development time frame and costs are substantially reduced when advancing a candidate into a clinical trial, which is possible without neglecting the comorbidities already associated with certain medications not to aggravate the patient condition provoked by the viral infection [6].

Several drugs that have been approved for cancer indication by the US FDA are now in COVID-19 clinical trials to test their efficiency in reducing mortality and speed up recovery. The following Table 1, Table 2, Table 3, Table 4, Table 5 and Table 6 represent anticancer drugs in clinical trials for COVID-19. In this review, we explore according to different categories of therapies which drugs represent more or fewer advantages for COVID-19. Appendix A is an updated list of all the anticancer drugs we could find or drugs used for the best supportive cancer care, which are being tested on their effectiveness to treat patients with mild to severe SARS-CoV-2.

### 5.1. Interferon-Based Therapies

The homeostasis maintained by the vascular endothelium in health is affected by COVID-19 infection. In clinical studies, patients with COVID-19 have higher levels of fibrinogen, fibrin degradation products, and D-dimer, which appear to be related to disease severity and thrombotic risk [19].

SARS-CoV-2 compromises the type 1 interferon antiviral response; therefore, IFN administration seemed a promising approach to stimulate macrophages, which engulf antigens and natural killer cells (NK cells). IFN might be able to strengthen the immune system by activating dormant components [20]. Clinical trials are running to test its effectiveness either alone or in combination with other drugs.

Ribavirin, lopinavir/ritonavir, remdesivir or hydroxychloroquine are some of the drugs tested in combination with IFNs in clinical trials (see Table 1). The study by Hung IF-N et al. demonstrated that early treatment with interferon beta-1b, lopinavir–ritonavir, and ribavirin is safe and highly effective in shortening the duration of the virus shedding, decreasing cytokine responses and allowing patients with mild to moderate disease to be discharged COVID-19 [21].

The problem is that when interferons boost the immune system, COVID-19 are likely to worsen before they improve. Giving anyone an interferon-based drug if they are still on a ventilator and their symptoms are about to overtake them may be fatal. This is why, in the case of viral infections, interferon therapies are usually only used as a last resort [22]. Nonetheless, interferon has already shown success against the antiviral activity, due to their ability to modulate the immune response, which is considered a “standard of care” in suppressing Hepatitis C and B infections [20].

### 5.2. Anticytokine Agents

The current COVID-19 infection is linked to elevated cytokine levels or hypercytokinemia. Patients who develop cytokine storms quickly experience cardiovascular collapse, multiple organ dysfunction and death [23]. The marked elevation of serum cytokines, especially tumor necrosis factor-alpha, interleukin 17 (IL-17), interleukin 8 (IL-8) and interleukin 6 (IL-6), is seen in patients with COVID-19 who go through pneumonia and hypoxia [24] (Table 2).

The administration of IL-6 blocking agents, such as tocilizumab and siltuximab, has been shown to be effective [25]. Repurposing tocilizumab would be interesting for the prevention or treatment of lung injury caused by COVID-19 since there is currently no effective antiviral therapy. In prospective studies, tocilizumab was linked to a lower relative risk of mortality, but the effects on other outcomes were inconclusive.

The drug siltuximab is a chimeric monoclonal antibody that binds to interleukin-6 (IL-6), preventing binding to soluble and membrane-bound interleukin-6 receptors. Current evidence showed that siltuximab led to a reduced mortality rate from COVID-19 promising to be a possible therapy; however, more studies are necessary [25].

### 5.3. Immune-Checkpoint Inhibitors

Immune checkpoints are regulatory molecules that are found on the surface of immune cells. When proteins on the surface of immune cells called T cells recognize and bind to partner proteins on other cells, such as tumor cells, immune checkpoints are activated. The T cells receive an “off” signal which may prevent cancer from being destroyed by the immune system. Therefore, immune checkpoint inhibitors are immunotherapy drugs that work by preventing checkpoint proteins from binding to their partner proteins. As a result, the “off” signal is not sent, allowing T cells to kill cancer cells [26,27].

The same principle can be applied for COVID-19 as a potential therapeutic approach (see Table 3). Evidence from preclinical models suggests that blocking programmed death receptor 1 (PD1) protects against RNA virus infections. Among the ICIs, antibodies capable of blocking the pathway of programmed death 1 (PD 1)/PD ligand-1 (PD L1) are promising. PD-1 expression levels on NK cells and T-cells were found to be highly upregulated in COVID-19 patients. When treated with anti-PD 1 and anti-PD L1 antibodies, they regain their T cell competence and effectively counteract viral infection [26,28]. Nivolumab and Pembrolizumab are ICIs that were successfully introduced into the management of various solid cancers, particularly for melanoma [24]. Currently, there is a phase II to trial to access efficacy for COVID-19. Pembrolizumab was tested in combination with tocilizumab [26].

### 5.4. Hormone Therapy

Androgen deprivation therapy (ADT), also known as androgen suppression therapy, is an antihormone therapy used to treat prostate cancer. Increasing evidence suggests that androgen has the potential to regulate the cellular TMPRSS2 expression and ACE2 [29].

TMPRSS2 is a membrane protease necessary for COVID pathogenesis, which is regulated by androgens. Blocking TMPRSS2 with bicalutamide can reduce viral replication and improve clinical outcomes. These agents may down-regulate TMPRSS2 mRNA and expression resulting in less entry of SARS-CoV-2 entry into cells and thus could arise as promising therapeutic tools in early SARS-CoV-2 infection and COVID-19 [30], see Table 4. A combination of bicalutamide in combination with camostat has the potential to reduce hospitalizations.

Toremifene used in the treatment of advanced breast cancer in postmenopausal women is a first-generation nonsteroidal-selective estrogen receptor modulator. It displays potential effects in blocking various viral infections, including MERS-CoV, SARS-CoV and Ebola virus. Prevents fusion between the viral and endosomal membrane by interacting with and destabilizing the virus membrane glycoprotein and eventually inhibiting viral replication [31]. Moreover, a preliminary study reveals a high potential for the synergistic effects of melatonin and toremifene to reduce viral infection and replication [32].

### 5.5. Inhibitor of Elongation Factor 1A and the Eukaryotic Initiation Factor 4A

Other molecules revealed potent pre-clinical efficacy against SARS-CoV-2 by inhibiting replication. In the life cycle of SARS-CoV-2, many host proteins play a role, and some are required for viral replication and translation. Drugs that target viral proteins are usually the focus of research, but a complementary approach is to target the required host proteins (Table 5).

Plitidepsin is an inhibitor of elongation factor 1A (eEF1A) and is an authorized drug in Australia for the treatment of multiple myeloma. Antiviral activity of plitidepsin has been analyzed in a human hepatoma cell line infected with the HCoV-229E-GFP virus, a virus similar to the SARS-CoV-2 virus [33]. Clinical studies using this drug are already taking place to assess safety and toxicity profile in patients with COVID-19 who require hospital admission, being the main goal is to select the recommended dose levels of plitidepsin for future phase 2/3 efficacy studies.

Another promising drug being tested in clinical trials is Zotatifin to assess its safety and tolerability. Zotatifin is a selective small-molecule inhibiting the eukaryotic initiation factor 4A (eIF4A), a powerful anti-proliferative target found at the intersection of the RAS and PI3K signaling pathways [34].

### 5.6. Blockade of Kinase Cascades

To test the hypothesis that PI3K blockade could hamper immune system hyperactivation and thus reduce lung inflammation and interfere with the viral cycle, researchers used one of the most successful targeted strategies in cancer treatment: kinase cascade blockade [35]. In a randomized placebo-controlled phase 2 study, Duvelisib, an orally bioavailable phosphatidylinositol 3-kinase (PI3K) selective inhibitor, is being evaluated for its ability to reduce inflammation in the lungs of patients with severe acute respiratory syndrome coronavirus 2 infections. As has been demonstrated repeatedly for multiple compounds in this pharmacological class, PI3K inhibitors, including the drug duvelisib, can cause lung inflammation and increase the risk of infections, and special caution is required during clinical trials using this class of molecules (Table 6).

On the other hand, Zanubrutinib is an irreversible Bruton tyrosine kinase inhibitor. The aberrant activation of the Bruton tyrosine kinase has a key role in the tumorigenesis of B-cell lymphoma. For COVID-19 evidence suggesting protective effects, a phase II trial is ongoing, aiming to reduce the disease-related immune dysregulation and hyper-inflammation [35].

### 5.7. Radiation and Prophylactic Vitamin D

Low-dose thoracic irradiation strategies with anti-inflammatory or prophylactic vitamin D have shown antiviral potential. However, there is a lack of direct pre-clinical and clinical evidence for COVID-19 and other therapeutics that may be more accessible, less risky, and less complicated for treatment [36].

Recently, we have acquired an unparalleled knowledge of the molecular processes and immune tolerance mechanisms regulating the occurrence and severity of human neoplasms, contributing to a wide variety of targeted anticancer and immunotherapy treatments [37]. Despite their specificity, however, small-molecule inhibitors and antibody-based therapies cause both on-and off-target effects, including immune-related pneumonia and diabetes, among other conditions, which need to be addressed when translating COVID-19 anticancer therapy. Now it is necessary to continue with clinical trials to overcome the uncertainties about the risks of certain therapeutics and understand which could be more beneficial in a time where vaccines are already available. Therapeutics along with immunization are the key to getting rid of the pandemic.

## 6. Conclusions

**Figure 1:**
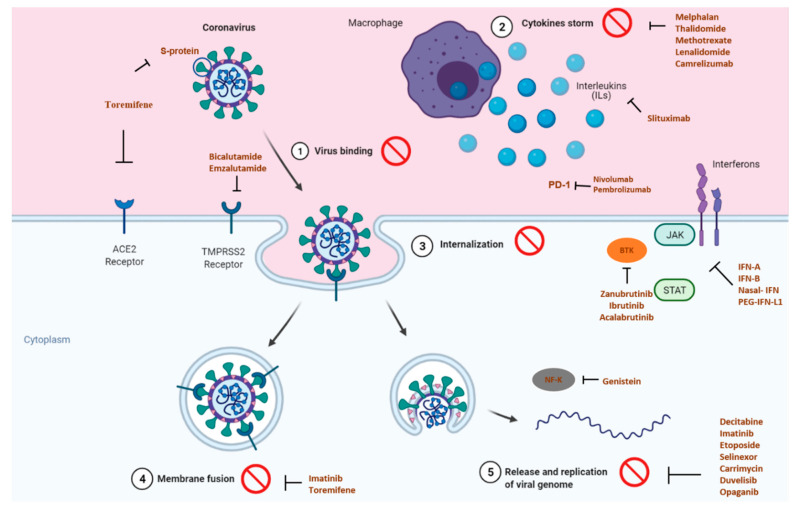
Principal targets of the anticancer drugs or drugs used for breast cancer supportive care, repurposed in clinical trials for COVID-19, adapted from BioRender templates [38].

The COVID-19 pandemic has swiftly swept through the world, resulting in huge morbidity and significant mortality. While the news of vaccination brings the promise to the end of the pandemic, the importance of medicines must not be forgotten since it helps to limit the spread of disease and allows both prevention and treatment. Either using repurposed drugs, alone or in combination or even new molecules, the pandemic provides an opportunity to create new models for evaluating novel therapeutic approaches quickly. Due to similarities between cancer and COVID-19, anticancer drugs are repurposed in clinical trials to test their efficacy in targeting inflammation, immune dysfunction, and coagulopathy. Figure 1 illustrates the principal targets of anticancer drugs repurposed in clinical trials for COVID-19.

Finally, the management of the SARS-CoV-2 pandemic includes multidisciplinary collaboration to identify suitable treatment options for everyone, including and especially countries with limited access to vaccines and people already hospitalized. From the evidence reviewed here, several anticancer drugs seem to retain a promising activity to treat patients with COVID-19.

## Figures and Tables

**Table 1:** Anticancer drugs in clinical trials for COVID-19: Interferon-based therapies.

Anticancer Drug	Viral—Host Targets	Mechanism of Action	Combination	Primary End-Point	Source (20 May 2021)
IFN	Jak1 and Tyk2	Jak1 and Tyk2	-	Negative SARS-CoV-2 RNA on a nasopharyngeal swab	[10]
	Jak1 and Tyk2	Jak1 and Tyk2	-		
IFN-B1A	Jak1 and Tyk2	Jak1 and Tyk2	-	Clinical Improvement	[10]
		Jak1 and Tyk2	Lopinavir, ritonavir	Percentage of subjects reporting severity	
			Hydroxychloroquine, lopinavir, ritonavir	Reduce Mortality	
			Hydroxychloroquine, lopinavir, ritonavir, umifenovir	Time to clinical improvement	
			Multifactorial	All-cause mortality	
IFN beta 1b	Jak1 and Tyk2	Jak1 and Tyk2	Remdesivir	Clinical improvement	[10]
			ribavirin	Reduce hospitalisation	
IFN-A2B	activate two Jak (Janus kinase) tyrosine kinases (Jak1 and Tyk2)	activate two Jak (Janus kinase) tyrosine kinases (Jak1 and Tyk2)	-	Improvement in FMTVDM Measurement with nuclear imaging	[10]
			-	Incidence of adverse events	
IFN-B1A/B	Jak1 and Tyk2	Jak1 and Tyk2	Hydroxychloroquine, lopinavir, ritonavir	Time to clinical improvement	[10]
IFN-B1B	Jak1 and Tyk2	Jak1 and Tyk2	Hydroxychloroquine, lopinavir, ritonavir	Time to negative NPS viral load	[10]
	Jak1 and Tyk2	Jak1 and Tyk2	Ribavirin, lopinavir, ritonavir	Time to negative NPS	

**Table 2:** Anticancer drugs in clinical trials for COVID-19: Anti-cytokine agents.

Anticancer Drug	Viral—Host Targets	Mechanism of Action	Combination	Primary End-Point	Source (20 May 2021)
Thalido-mide	Inhibition of inflammatory cytokine production	Inhibit the producti-on of interleukin (IL)-6	-	Time to clinical recovery	[10]
Siltuximab	Interleukin-6	Interleukin-6	-	The proportion of patients Requiring ICU admission at any time	
			-	Mortality in siltuximab treated patients	[11]
			Anakinra	Time to clinical improvement	
			tocilizumab	Ventilator-free days	

**Table 3:** Anticancer drugs in clinical trials for COVID-19: Immune-checkpoint inhibitors.

Anticancer Drug	Viral—Host Targets	Mechanism of Action	Combination	Primary End-Point	Source (20 May 2021)
PD-1 blocking antibody	PD-1	Can prevent the tumor cell from binding PD-1	-	Lung injury score	[10]
Nivolumab	PD-1/PD-L1 pathway blockade	Immune homeostasis restoration	-	Time to clinical improvement	[10]
			-	Efficacy and safety	[10]
			-	Viral clearance kinetics	[10]
Pembrolizumab	PD-1/PD-L1 pathway blockade	Immune homeostasis restoration	Tocilizumab	Percentage of patients with the normalisation of SpO_2_ ≥96% in room air	[10]

**Table 4:** Anticancer drugs in clinical trials for COVID-19: Hormone therapy.

Anticancer Drug	Viral—Host Targets	Mechanism of Action	Combination	Primary End-Point	Source (6 February 2021)
Bicalutami-de	Downregulates TMPRSS2	Binding of androgen receptor	-	COVID-19 symptom relief	[9]
			Camostat	Reduce number of participants requiring hospitalization	
Enzalutami-de	Reduce androgen driven morbidity in COVID-19	Competitive binder of androgens	-	Time to worsening of disease	[9]
Toremifene	Interaction with coronavirus proteins	Inhibition of viral membranes fusion with Host cell endosomes	Melatonin	Clinical improvement	[10]
Tamoxifen	Decreased the PGE2 production	Compete with 17β-estradiol (E_2_) at the receptor site	-	Lung injury score	[9]

**Table 5:** Anticancer drugs in clinical trials for COVID-19: The inhibitor of elongation factor 1A and the eukaryotic initiation factor 4A.

Anticancer Drug	Viral—Host Targets	Mechanism of Action	Combination	Primary End-Point	Source (20 May 2021)
Plitidepsin	Blockade of eEF1A	Interference with the viral cycle	-	Frequency of occurrence of Grade 3 or higher AEs	[10]
Zotatifin	Blockade of eIF4A	Inhibition of protein biogenesis	-	–	[10]

**Table 6:** Anticancer drugs in clinical trials for COVID-19: Blockade of kinase cascades.

Anticancer Drug	Viral—Host Targets	Mechanism of Action	Combination	Primary End-Point	Source (20 May 2021)
Duvelisib	PI3K inhibition	Immune homeostasis restoration and viral replication inhibition	-	Overall survival	[10]
			-	Reduce overall necessity of ventilation	
Zanubrutinib	Inhibition of the Bruton tyrosine kinase	Protection against immune, lethal and sepsis-induced pulmonary injuries	-	The respiratory failure-free survival rate	[10]
Carrimycin	Inhibit the replication of SARS-CoV-2 in the cells	Inhibits mTOR pathway	-	Fever to normal time	[11]
			-	Percentage of patients alive without the need for supplemental oxygen and ongoing in patient-medical care	
Ibrutinib	Inhibition of the Bruton tyrosine kinase	Protection against immune-induced lung injury	-	The respiratory failure-free survival rate, overall survival	[10]
			-	Patients with diminished respiratory failure and death	

## Data Availability

Not applicable.

## Abbreviations

Coronavirus disease 2019 (COVID-19); severe acute respiratory syndrome coronavirus-2 (SARS-CoV-2); acute respiratory distress syndrome (ARDS); world health organization (WHO); the food and drug administration (FDA); interferons (IFNs); transmembrane-serine-protease-2 (TMPRSS2); angiotensin-converting enzyme 2 (ACE2); phosphatidylinositol 3-kinase (PI3K);elongation factor 1A (eEF1A); eukaryotic initiation factor 4A; the eukaryotic initiation factor 4A (eIF4A);programmed death receptor 1 (PD1); programmed death ligand-1 (PD L1); immune-checkpoint inhibitors (ICIs); tumor necrosis factor alpha (TNF-α);interleukin 17 (IL-17); interleukin 8 (IL-8);interleukin 6 (IL-6); Janus kinase (Jak); tyrosine kinases (Tyk); nucleocapsid (N); spike (S); membrane (M); envelope (E); endoplasmic reticulum (ER); endoplasmic reticulum-Golgi intermediate compartment(ERGIC); natural killer (NK)

## Appendix A

**Table A1:** List of drugs with anticancer effects or used for best supportive cancer care, in clinical studies for the treatment of COVID-19.

Anticancer Drug	Viral—Host Targets	Mechanism of Action	Tested in Clinical Trials	NCT Identifier	Combination	Status	Phase	Eligible Population	Primary End-Point	Source (20 May 2021)
Acalabrutinib	BTK	Inhibits the activity of BTK and prevents the activation of the B-cell antigen receptor	United states	NCT04380688	-	Completed	Phase 2	COVIDCOVID-19 infection	Occurrence of Adverse Events and Serious Adverse Events	[10]
			Several locations	NCT04346199	-	Completed	Phase 2	COVID-19 infection	Subject alive and free of respiratory failure	
Bevacizumab	VEGF	Vascular permeability inhibition	Yes (France and China)	NCT04275414	-	Completed	Phase 2	Severe lung disease or critical disease	Change of PaO_2_ to FiO_2_ ratio	[10]
				NCT04344782	-	Not yet recruiting	Phase 2	Severe lung disease	Number of patients who avoid mechanical-assisted ventilation	
				NCT04305106	-	Recruiting	Not applicable	Disease requiring O_2_-support	Time to clinical improvement	
Bicalutamide	Downregulates TMPRSS2	Binding of androgen receptor	Yes	NCT04509999	-	Recruiting	Phase 3	COVID-19 infection, confirmed	COVID-19 symptom relief	[9]
				NCT04652765	camostat	Recruting	Phase 1	COVID-19 infection, confirmed	Reduce number of participants requiring hospitalization	
Camrelizumab	Immune homeostasis	PD-1/PD-L1 pathway	Yes (China)	ChiCTR2000029806	-	Recruiting	Not applicable	COVID-19 infection	Proportion of patients with	[10]
		Blockade							a lung injury score	
		-							reduction	
Carrimycin	Inhibit the replication of SARS-CoV-2 in the<break/>cells	Inhibits mTOR *pathway*	Not provided	NCT04286503	-	Not yet recruting	Phase 4	COVID-19 infection	Fever to normal time	[11]
				NCT04672564	-	Recruting	Phase 3	Patient with SARS-CoV-2 infection	Percentage of patients alive without need for supplemental oxygen and ongoing in patient-medical care	
Decitabine	Nucleic Acid Synthesis Inhibitor	Nucleic acid synthesis inhibitor	Yes (USA)	NCT04482621	-	Recruting	Phase 2	COVID infection	Clinical improvement	[9]
Duvelisib	PI3K inhibition	Immune homeostasis restoration and viral replication inhibition	Yes (USA)	NCT04372602	-	Recruting	Phase 2	Critical disease	Overall survival	[10]
				NCT04487886	-	Recruting	Phase 2	severe COVID-19 who do not require mechanical ventilation	Reduce overall necessity of ventilation	
Ensifentrine	High selectivity for PDE3 and PDE4 over other enzymes and receptors to minimize off-target effects	Dual inhibitor of phosphodiesterase 3 (PDE3) and 4 (PDE4)	United states	NCT04527471	None	Active, not recruting	Phase 2	SARS-CoV-2 infection	Proportion of patients with recovery	[11]
Enzalutamide	reduce androgen driven morbidity in COVID-19	Competitive binder of androgens	Sweden	NCT04475601	-	Recruting	Phase 2	SARS-CoV-2 infection	Time to worsening of disease	[9]
Etoposide	Topoisomerase II	Inhibits DNA synthesis by forming a complex with topoisomerase II and DNA	United states	NCT04356690	-	Active, not yet Recruting	Phase 2	Confirmed COVID-19 infection	Change in pulmonary status	[9]
FN-B1A	Jak1 and Tyk2	Jak1 and Tyk2	UK	NCT04385095	-	Recruiting	Phase 2	COVID-19 infection	Clinical Improvement	[10]
		Jak1 and Tyk2	Several locations	NCT04315948	Lopinavir, ritonavir	Active, not yet Recruiting	Phase 3	COVID-19 infection	Percentage of subjects reporting severity	
		-	Iran	NCT04350671	Hydroxychloroquine, lopinavir, ritonavir	Enrolling by invitation	Phase 4	COVID-19 infection	Reduce Mortality	
		-	Irna	NCT04350684	Hydroxychloroquine, lopinavir, ritonavir, umifenovir	Enrolling by invitation	Phase 4	COVID-19 infection	Time to clinical improvement	
		-	Several locations	NCT02735707	Multifactorial	Recruiting	Phase 4	COVID-19 infection	All-cause mortality	
Genistein	Inhibition of<break/>both transcription nuclear factor-κB (NF-κB) activation and chemokine-8 secretion	Triggers the ER stress through upregulation of glucose-regulated protein 78 (GRP78) expression	United statwa	NCT04482595	-	Recruting	Phase 2	Patients hospitalized for COVID-19	Change in Diffusing capacity of the lungs for carbon monoxide	[9]
Ibrutinib	Inhibition of the Bruton tyrosine kinase	Protection against immune-induced lung injury	Yes (USA)	NCT04375397	-	Active, not yet Recruiting	Phase 2	Hospitalised patients with severe pneumonia	Respiratory failure-free survival rate, overall survival	[10]
				NCT04439006	-	Recruting	Phase 2	Patients Requiring Hospitalization	Patients with diminished respiratory failure and death	
IFN	Jak1 and Tyk2	Jak1 and Tyk2	Canada	NCT04354259	-	Recruiting	Phase 2	COVID-19 infection	Negative SARS-CoV-2 RNA on nasopharyngeal swab	[10]
	Jak1 and Tyk2	Jak1 and Tyk2	China	NCT04331899	-	Active, Not yet recruiting	Phase 2	COVID-19 infection		
IFN beta 1b	Jak1 and Tyk2	Jak1 and Tyk2	Hong kong	NCT04647695	Remdesivir	Recruiting	Phase 2	high risk of clinical deterioration	Clinical improvement	[10]
				NCT04494399	ribavirin	Recruiting	Phase 2	COVID-19 infection	Reduce hospitalisation	
IFN-A2B	activate two Jak (Janus kinase) tyrosine kinases (Jak1 and Tyk2)	activate two Jak (Janus kinase) tyrosine kinases (Jak1 and Tyk2)	United States	NCT04349410	-	Completed	Phase 2/3	CoVid-19 infection	Improvement in FMTVDM Measurement with nuclear imaging	[10]
				NCT04379518	-	Recruiting	Phase 1/2	Patients with cancer and mild or moderate symptomatic infection	Incidence of adverse events	
IFN-B1A/B	Jak1 and Tyk2	Jak1 and Tyk2	Irnan	NCT04343768	Hydroxychloroquine, lopinavir, ritonavir	Completed	Phase 2	COVID-19 infection	Time to clinical improvement	[10]
IFN-B1B	Jak1 and Tyk2	Jak1 and Tyk2	Hong kong	NCT04350281	Hydroxychloroquine, lopinavir, ritonavir	Completed	Phase 2	COVID-19 infection	Time to negative NPS viral load	[10]
	Jak1 and Tyk2	Jak1 and Tyk2	Hong kong	NCT04276688	Ribavirin, lopinavir, ritonavir	Completed	Phase 2	COVID-19 infection	Time to negative NPS	
Imatinib	BCR/ABL kinase inhibition	Blockade of cell entry and endosomal trafficking	Yes (France, Spain and USA)	NCT04357613	-	Not yet Recruitng	Phase 2	Hospitalised patients	Rate of prevented severe disease worsening	[10]
Interferon	Jak1 and Tyk2	Jak1 and Tyk2	China	NCT04291729	Danoprevir, ritonavir	Completed	Phase 4	COVID-19 infectio	Rate of composite adverse outcome	[10]
Lenalidomide	Immunomodulatory agent	substrate specificity of the CRL4^CRBN^ E3 ubiquitin ligase	Spain	NCT04361643	-	Not yet recruting	Phase 4	COVID-19 infection	Clinical improvement	[10]
Leronlimab	Disruption of the CCL5/RANTES-CCR5 pathway	Immune homeostasis restoration	Yes (USA)	NCT04343651	-	Active, not recruitng	Phase 2	Mild/moderate disease	Clinical improvement	[10]
				NCT04347239	-	Recruting	Phase 2	Severe lung disease or critical disease	Overall survival	[10]
Masitinib	directly binds to the active site of 3CLpro	Tyrosine kinase inhibitor	Yes (France)	NCT04622865	Isoquercetin	Recrutiting	Phase 2	COVID 19 diagnosis	Clinical status of patients at day-15	[9]
Melphalan	anti-inflammatory response	Inhibition of DNA and RNA synthesis by realizing an alkylating peptide	Yes (Russian Federation)	NCT04380376	-	Recrutiting	Phase 2	COVID 19 diagnosis	The changes of COVID Ordinal Outcomes Scale	[9]
Methotrexate	Immunomodulatory agent	inhibition of folate dependent pathways leading to inhibition of DNA synthesis	France	NCT04481633	Hydroxychloroquine	Recruiting	Not applicable	COVID-19 infection	Rate of patients with positive anti-COVID19 serology	[10]
Nasal IFN-A1B	Jak1 and Tyk2	Jak1 and Tyk2	China	NCT04320238	Anti-thymosin	Recruiting	Phase 3	Formally serving medical staff in Taihe Hospital	new-onset COVID-19	[10]
Nivolumab	PD-1/PD-L1 pathway blockade	Immune homeostasis restoration	Yes (France and China)	NCT04343144	-	Not yet recruiting	Phase 2	Disease requiring O_2_-support	Time to clinical improvement	[10]
				NCT04413838	-	Not yet recruiting	Phase 2	Obese individuals	Efficacy and safety	[10]
				NCT04356508	-	Not yet recruiting	Phase 2	Clinically stable patients with mild or moderate disease and asymptomatic patients	Viral clearance kinetics	[10]
Opaganib	Inhibition of sphingosine kinase-2	Anti-inflammatory and antiviral properties	Yes (Israel)	NCT04414618	-	Completed	Phase 2	Disease requiring O_2_-support	Measurement of the daily O_2_ requirements	[10]
				NCT04467840	-	Recruting	Phase 2 and 3	Disease requiring O2-support	Reduce Intubation and mechanical ventilation	
				NCT04435106	-	Completed	-	severe COVID-19 who required oxygen support via high-flow nasal cannula	Measure the time to weaning from high-flow nasal cannula and	
									Measure the time to breathing ambient	
				NCT04502069	-	Withdrawn (To be replaced with a randomized placebo-controlled study.)	Phase 1 and 2	Pneumonia Requiring Oxygen	Time to breathing room air	
PD-1 blocking antibody	PD-1	Can prevent the tumor cell from binding PD-1	Not provided	NCT04268537	-	Not yet recruiting	Phase 2	COVID-19 infection	lung injury score	[10]
Peg-IFN-L1	Jak1 and Tyk2	Jak1 and Tyk2	United states	NCT04343976	-	Enrolling by invitation	Phase 2	COVID-19 infectio	Negative SARS-CoV-2 RNA on nasopharyngeal swab	[10]
Peg-IFN-L1A	Jak1 and Tyk2	Jak1 and Tyk2	United states	NCT04388709	-	Withdrawn (Due to the number of competing trials at their site, the study team has closed enrollment and withdrawn this trial.)	Phase 2	COVID-19 infection	Number of participants with resolution of hypoxia	[10]
			United states	NCT04344600	-	Not yet recruiting	Phase 2	COVID-19 infection	Proportion of participants with no evidence of SARS-CoV-2 infection	
Pembrolizumab	PD-1/PD-L1 pathway blockade	Immune homeostasis restoration	Yes (Spain)	NCT04335305	Tocilizumab	Recruiting	Phase 2	Severe lung disease or critical disease	Percentage of patients with normalisation of SpO_2_ ≥96% on room air	[10]
Plitidepsin	Blockade of eEF1A	Interference with the viral cycle	Yes (Spain)	NCT04382066	-	Completed	Phase 1	Hospitalised patients	Frequency of occurrence of Grade 3 or higher AEs	[10]
Selinexor	Blockade of nucleocytoplasmic transport	Antiviral and anti-inflammatory properties	Yes (USA, France, Austria, Spain and United Kingdom)	NCT04355676	-	Withdrawn (No participants enrolled)	Phase 2	Hospitalised patients with moderate or severe disease	Percentage of participants with at least a two-point improvement in the ordinal scale	[10]
				NCT04349098	-	Completed	Phase 2	Hospitalised patients with severe disease	Improvement	
				NCT04534725	-	Recruiting	Phase 3	received cancer related treatment	COVID-19 Prevention and Treatment in Cancer	
SFX-01	up-regulates the Nrf2 pathway	Up-regulates the Nrf2 pathway	UK + Evgen Pharma	-	-	Enrolment begins in July (results are expected in 2021)	Phase 2/3	-	Efficacy at treating ARDS	[11]
Siltuximab	Interleukin-6	Interleukin-6	Spain	NCT04329650	-	Recruiting	Phase 2	Hospitalized patient	Proportion of patients requiring ICU admission at any time	[11]
			Italy	NCT04322188	-	Completed	-	COVID-19 infection	mortality in siltuximab treated patients	
			Belgium	NCT04330638	Anakinra	Active, not recruting	Phase 3	COVID-19 infection	Time to Clinical Improvement	
			Saudi Arabia	NCT04486521	tocilizumab	Recruiting	-	COVID-19 infection	Ventilator-Free Days	
Tamoxifen	Decreased the PGE2 production	Compete with 17β-estradiol (E_2_) at the receptor site	Egypt	NCT04568096	-	Not yet recruting	Phase 2	Adult SARI patients with COVID-19 infection	Lung injury score	[9]
Tetrandrine	Ability to block the two-pore channel 2 (TPC2)	Checkpoint inhibitor of the cell cycle	China	NCT04308317	-	Enrolling by invitation	Phase 4	COVID-19 infection	Survival rate	[10]
Thalidomide	inhibition of inflammatory cytokine production	Inhibit the production of interleukin (IL)-6,	China	NCT04273529	-	Not yet recruiting	Phase 2	COVID-19 infection	Time to Clinical recovery	[10]
Toremifene	Interaction with coronavirus proteins	Inhibition of viral membranes fusion with host cell endosomes	? (Not provided)	NCT04531748	Melatonin	Withdrawn (Funding)	Phase 2	-	Clinical improvement	[10]
Zanubrutinib	Inhibition of the Bruton tyrosine kinase	Protection against immune, lethal and sepsis-induced pulmonary injuries	Yes (USA)	NCT04382586	-	Completed	Phase 2	Disease requiring O_2_-support	Respiratory failure-free survival rate	[10]
Zotatifin	Blockade of eIF4A	Inhibition of protein biogenesis	No	NCT04632381	-	Not yet recruiting	Phase 1	-	-	[10]
